# Antiproliferative Effects of 1α-OH-vitD_3_ in Malignant Melanoma: Potential Therapeutic implications

**DOI:** 10.1038/srep40370

**Published:** 2017-01-11

**Authors:** Lucia Spath, Alessandra Ulivieri, Luca Lavra, Laura Fidanza, Marta Carlesimo, Maria Giubettini, Alessandra Narcisi, Emidio Luciani, Barbara Bucci, Daniela Pisani, Salvatore Sciacchitano, Armando Bartolazzi

**Affiliations:** 1 Pathology Research Laboratory, Sant’Andrea University Hospital, via di Grottarossa 1035, 00189 Rome, Italy; 2 Laboratory of Biomedical Research, Niccolò Cusano University Foundation, via Don Carlo Gnocchi 3, 00166 Rome, Italy; 3 Dermatology Unit, Sant’Andrea University Hospital, via di Grottarossa 1035, 00189 Rome, Italy; 4 Internal Medicine Sant’Andrea University Hospital, via di Grottarossa 1035, 00189 Rome, Italy; 5 Molecular and Cellular Tumor Pathology Laboratory, Cancer Center Karolinska, Karolinska Hospital, CCK R8:04, S-17176, Stockholm, Sweden

## Abstract

Early detection and surgery represent the mainstay of treatment for superficial melanoma, but for high risk lesions (Breslow’s thickness >0.75 mm) an effective adjuvant therapy is lacking. Vitamin D insufficiency plays a relevant role in cancer biology. The biological effects of 1α hydroxycholecalciferol on experimental melanoma models were investigated. 105 melanoma patients were checked for 25-hydroxycholecalciferol (circulating vitamin D) serum levels. Human derived melanoma cell lines and *in vivo* xenografts were used for studying 1α-hydroxycholecalciferol-mediated biological effects on cell proliferation and tumor growth. 99 out of 105 (94%) melanoma patients had insufficient 25-hydroxycholecalciferol serum levels. Interestingly among the six with vitamin D in the normal range, five had a diagnosis of *in situ*/microinvasive melanoma. Treatment with 1α-hydroxycholecalciferol induced antiproliferative effects on melanoma cells in *vitro* and *in vivo,* modulating the expression of cell cycle key regulatory molecules. Cell cycle arrest in G1 or G2 phase was invariably observed in vitamin D treated melanoma cells. The antiproliferative activity induced by 1α-hydroxycholecalciferol in experimental melanoma models, together with the discovery of insufficient 25-hydroxycholecalciferol serum levels in melanoma patients, provide the rationale for using vitamin D in melanoma adjuvant therapy, alone or in association with other therapeutic options.

Cutaneous melanoma represents 5–7% of all skin malignancies, but it is responsible for about 75% of deaths from skin tumors. The incidence of melanoma is increasing at an alarming rate, with a lifetime risk of developing a melanoma of 1/58 for USA males and 1/25 for Australian males^1^. Although early detection and surgery represent the mainstay of treatment for localized lesions, no effective therapy for metastatic melanoma is available so far. Furthermore patients with high risk melanomas (Breslow thickness >0.75 mm) are still orphans of an effective adjuvant therapy^2^,^3^,^4^,^5^,^6^. Vitamin D, also worded D-hormone, has pleiotropic effects of relevance to cancer, which includes regulation of cell growth and differentiation, induction of apoptosis and regulation of tumor/immune-system interactions^7^,^8^,^9^,^10^,^11^.

Interestingly a strong association between low serum levels of 25-hydroxycholecalciferol (25-OH-vitD_3_) and increased cancer incidence and cancer–related mortality has been demonstrated in large population studies^12^,^13^. Several clinical studies and meta-analysis of case-control and cohort studies also support this picture^14^,^15^,^16^. Furthermore, preclinical studies indicate that active metabolites of vitamin D or their synthetic derivatives have potential anticancer activity and could be used to potentiate the anticancer effects of several cytotoxic and antiproliferative drugs^17^,^18^,^19^,^20^. Vitamin D-mediated-biological effects require the expression of vitamin D receptor (VDR) on the target cells and the integrity of downstream effectors, among which 1α-hydroxylase (1α-OHase) and 25-hydroxylase (25-OHase) are necessary for the synthesis of vitamin D active metabolites, whereas 24-hydroxylase (24-OHase) regulates vitamin D inactivation and catabolism (Supplementary Figure S1). Alterations of VDR receptor and/or downstream enzymatic repertoire may potentially impair vitamin D responsivity of the target cells^21^,^22^. The aim of this experimental study was to analyze the biological effects induced by 1α-hydroxycholecalciferol (1α-OH-vitD_3_) in malignant melanoma models *in vitro* and *in vivo.* A panel of primary melanoma cell lines derived from patients with metastatic disease were found to constitutively express functional VDR, 25-OHase and 1α-OHase, indicating a potential vitamin D sensitivity. Treatment of melanoma cells with 1α-OH-vitD_3_ strongly impaired cell proliferation *in vitro* and tumor growth *in vivo.* Moreover low serum levels of 25-OH-vitD_3_ (10–30 ng/mL) were discovered to occur in the large majority melanoma patients (all stages) at time of first diagnosis^23^.

All together these findings open a clinical relevant question on the possibility to use vitamin D or its active metabolites in melanoma adjuvant therapy, in particular for high risk lesions, for which an observational approach is currentlty adopted.

## Results

### Insufficient 25-OH-vitamin-D_3_ Serum Levels is a Common Feature of Melanoma Patients at time of first diagnosis

**Figure 1:**
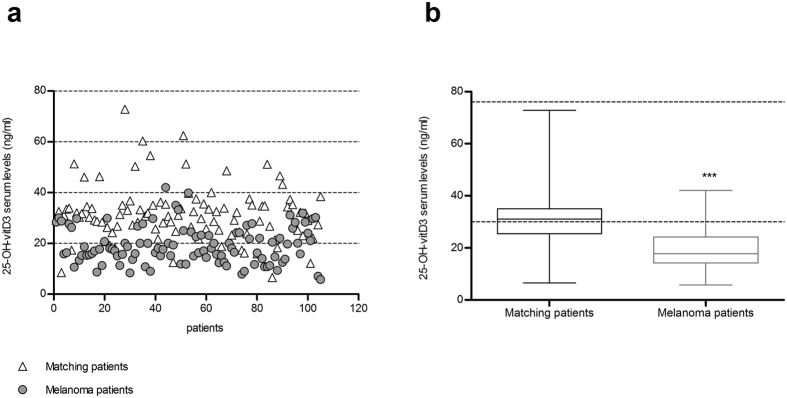
Insufficient 25-OH-vitD serum level is a common feature in melanoma patients. (**a**) Comparative evaluation of 25-OH-vitD serum levels registered in 105 melanoma patients at time of surgery (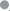) and in 101 matched (for sex and age) healthy individuals (∆). (**b**) The area between 30–76 ng/mL, which represents 25-OH-vitD sufficiency, is marked by a gray line. Standard deviation is shown (*Statistical analysis using t-test:* ***p value < 0.005).

Evaluation of 25-OH-vitD_3_ serum levels was performed on 105 melanoma patients (unselected) before surgery. Surprisingly, 99 out of 105 patients (94%) showed deficient or insufficient 25-OH-vitD_3_ serum levels, with values ranging from 7 to 30 ng/mL. Normal serum levels of 25-OH-vitD_3_ (30–76 ng/mL) were detected in 6 patients only (6% of the cases), 5 of which bearing *in situ* or micro-invasive melanoma and one a nodular melanoma with lymph node metastasis (Supplementary Table S1 and Fig. 1a,b). Data regarding basal serum levels of 25-OH-vitD_3_ in 101 matched (for sex and age) blood donors without evidence of neoplastic or chronic diseases were obtained from the Sant’Andrea Hospital data bank and used as comparative control. In this cohort, 55 subjects out of 101 (54%) had 25-OH-vitD_3_ serum levels in the normal range. The observed difference was statistically significant (p < 0.005).

The expression of vitamin D receptor (VDR) could be a critical event for vitamin D sensitivity. As demonstrated in RT-PCR the panel of melanoma cell lines considered in this study invariably expressed VDR and the complete repertoire of transcripts that is required for vitamin D activation and catabolism, namely 25-hydroxylase (gene CYP27A1), 1α-hydroxylase (gene CYP27B1) and 24-hydroxylase (gene CYP24A1) (see supplementary Figure S2A). The expression of VDR was also confirmed in western blot and immunocytochemistry for those melanoma cells used for establishing *in vitro* and *in vivo* experimental models to work with (Supplementary Figure S2B and data not shown).

The presence of VDR polymorphism was also investigated in a panel of melanoma cell lines used for experimental procedures (Supplementary Table S2). As expected, the identified polymorphisms did not impair VDR function. In fact all melanoma cell lines used in this study showed an antiproliferative response to 1α-OH-vitD_3_ *in vitro* (see below).

### 1α-Hydroxycholecalciferol and Vitamin D_2_ Active Derivative Paricalcitol Inhibit Melanoma Cell Proliferation *in Vitro*

**Figure 2:**
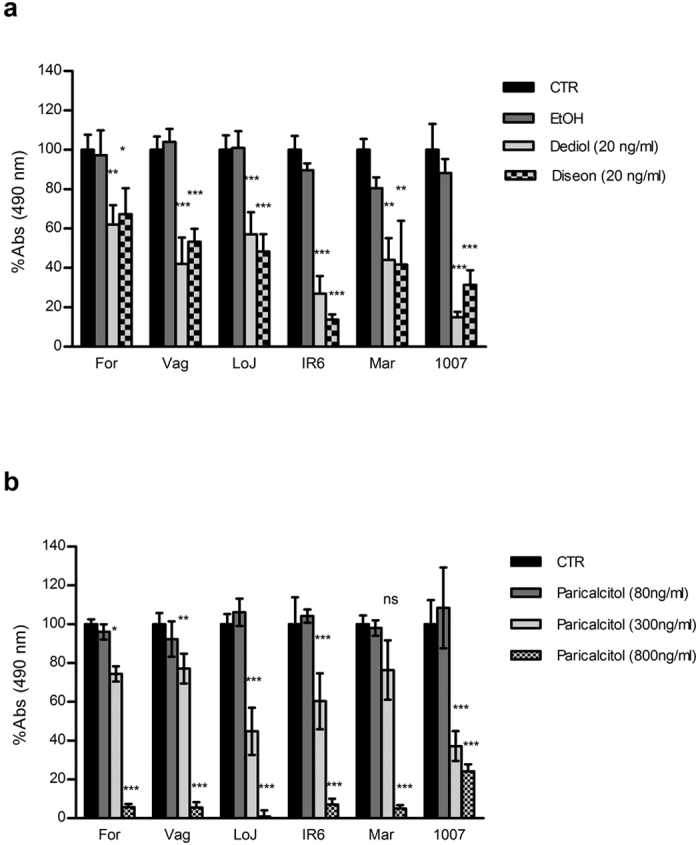
Antiproliferative effects of 1α-hydroxycholecalciferol and vitamin-D_2_ synthetic derivative paricalcitol on melanoma cells *in vitro.* Melanoma cells proliferation as determined by colorimetric tetrazolium salt assay (MTS assay) in the presence of (**a**) 20 ng/mL (5 × 10^8^ M) 1α-hydroxycholecalciferol (two different pharmaceutical preparations) and (**b**) vitamin-D_2_ active derivative paricalcitol at concentration ranging from 80 ng/mL to 800 ng/mL. Six metastatic melanoma cell lines were used in this experiment. The assay was performed after 72 hours of vitamin-D treatment. Untreated cells (CTR) and cells incubated with ethanol (EtOH 0.1%) were used as controls. *(Experiments in triplicate. S.D. is reported on the top of each column. Statistical analysis using ANOVA and Bonferroni post-hoc test: ns* = not significant; *p < 0.05; **p < 0.01; ***p < *0.001*).

Six well-characterized melanoma cell lines were used for studying the biological effects induced by 1α-OH-vitD_3_ on melanoma growth *in vitro*. Cells were cultured in the presence or absence of 1α-OH-vitD_3_ 20 ng/mL (5 × 10^−8^ M). Two different pharmaceutical preparations of this active 1α-OH-vitD_3_ metabolite (Diseon ^®^ and Dediol ^®^) were used for these experiments. 1α-OH-vitD_3_ consistently impaired melanoma cell proliferation *in vitro* and such a biological effect was measurable after 72 hrs of treatment (Fig. 2a).

**Figure 3:**
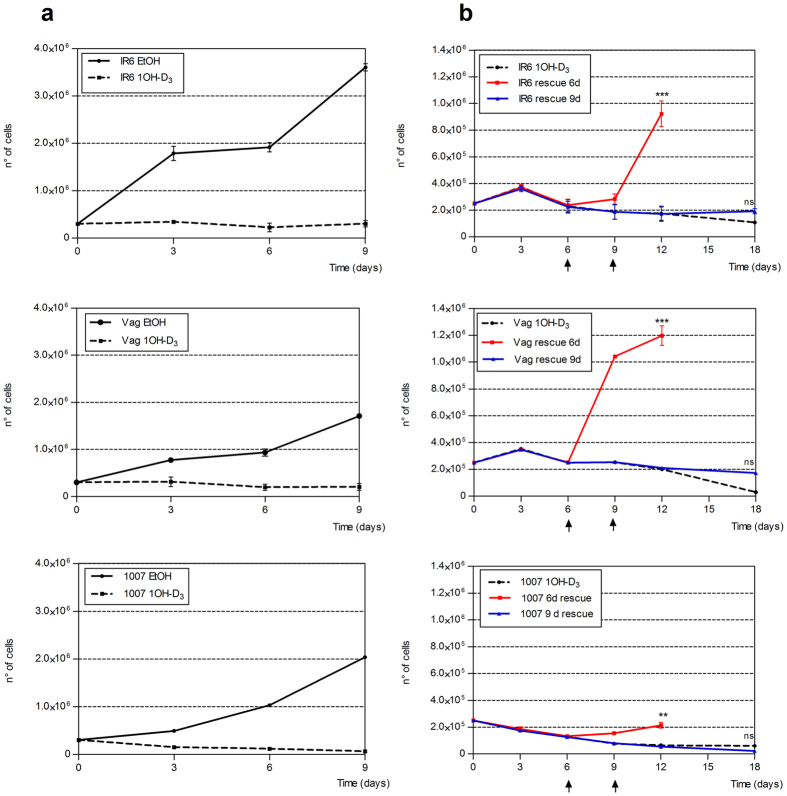
Effects of long-term incubation with 1α-hydroxycholecalciferol on tumorigenic melanoma cell lines. (**a**) Melanoma cell proliferation analyzed by cell counting after 3, 6 and 9 days of 1α-OH-D_3_ treatment, is strongly inhibited in the presence of vitamin-D_3_. This effect was not observed in melanoma control cells growing in standard vitD_3_ –free medium (solvent alone). (**b**) Proliferation assay was performed after 1α-OH-vitD_3_ withdrawal at 6 and 9 days. As showed in the right panel, cell proliferation was rescued up to 6 days of treatment. For longer vitD_3_ treatment (>9 days) all the melanoma cells failed to be recovered. *(Experiments in triplicate. Mean* ± S.D. is reported. Statistical analysis using ANOVA and Bonferroni post-hoc test: **p < 0.01; ***p < 0.001).: ** p < 0.01; ***p < *0.001*.

Similar experiments were performed with the synthetic active derivative of vitamin D_2_ named paricalcitol. In this case a concentration of 0.8 μg/mL was required to consistently inhibit melanoma cell proliferation *in vitro* (Fig. 2b). Tumorigenic melanoma cell lines VAG, IR6 and 1007 were then selected for establishing melanoma xenografts for a new set of experiments *in vivo*. Cells were first cultured in the presence or absence of 1α-OH-vitD_3_ 20 ng/mL (5 × 10^−8^ M) for 9 days. Once again inhibition of melanoma cell proliferation was observed after few days of 1α-OH-vitD_3_ treatment (Fig. 3a). To better understand whether the vitamin D-dependent inhibition of melanoma cell growth was a reversible event, a longer vitamin D treatment (18 days) was considered and a proliferation assay was finally performed after 1α-OH-vitD_3_ withdrawal. As shown in Fig. 3b cell proliferation was recoverable up to 6 days of treatment, while for longer treatment (>9 days) melanoma cells failed to be recovered in a vitamin D –free medium. This biological effect was statistically significant (p < 0.001).

**Figure 4:**
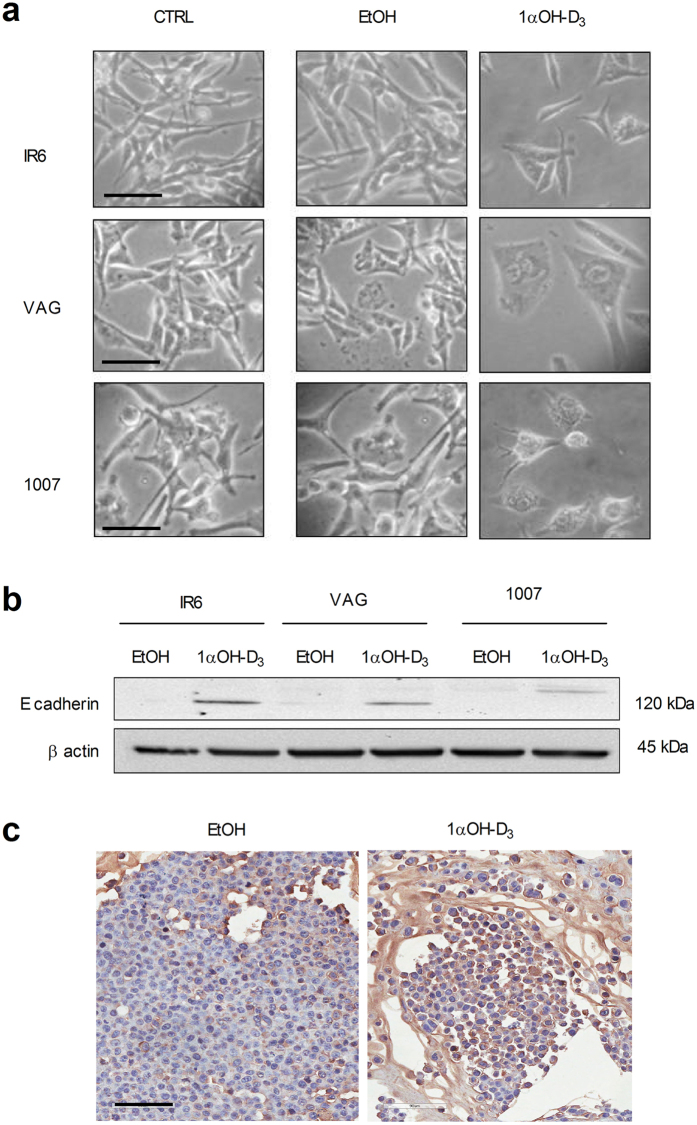
1α-hydroxycholecalciferol–mediated effects on cell morphology and differentiation. (**a**) Morphological aspects of melanoma cell differentiation observed after 18 days of vitamin-D_3_ treatment. Melanoma cells cultured in conventional vitamin-D_3_ free medium (CRT) and medium supplemented with solvent alone (0.1% ethanol) were used as comparative controls. Note single scattered IR6, VAG and 1007 melanoma cells with enlarged cytoplasm and small dendritic processes after 18 days of vitamin-D_3_ treatment (scale bar = 100 μm). (**b**) E-cadherin increased expression in vitamin-D_3_ treated cells, as evaluated in western blot analysis by using a specific mAb; The slight differences in MW of the observed bands are likely due to the expression of cell-specific isoforms of the protein. β-actin was used as loading control (figure derived from cropped gel/blot for semplification). (**C**) E-chaderin expression at immunohistochemical level on vitamin-D_3_ treated and untreated representative melanoma xenografts (post-autopsy) (indirect immunoperoxidase; scale bar = 200 μm).

Interestingly, melanoma cells exposed to vitamin-D failed to growth efficiently *in vitro,* but this effect was not related to cell death. Morphological changes consisting in cytosol enlargement with appearance of small dendritic processes were invariable observed in vitamin D treated melanoma cells at the end of the experiments, together with an increased expression of E-cadherin (Fig. 4a–c). The slight differences in the mobility of E-chaderin molecular species observed in western blot analysis are likely due to cell-specific post-translational modifications and/or to the occurence of alternative splicing (expression of different isoforms) as already reported in the literature^24^. These findings support the evidence that 1α-OH-vitD_3_ impairs melanoma cell proliferation and triggers differentiation^7^.

To deeply investigate the anti-proliferative effects induced by 1α-OH-vitD_3_ on melanoma cells *in vitro*, DNA content and cell cycle distribution of 1α-OH-vitD_3_-treated and untreated cell lines were analysed. Cytofluorimetric analysis was then performed after treatment of the cell lines with 1α-OH-vitD_3_ 20 ng/ml (5 × 10^−8^ M) at 3, 6 and 9 days.

**Figure 5:**
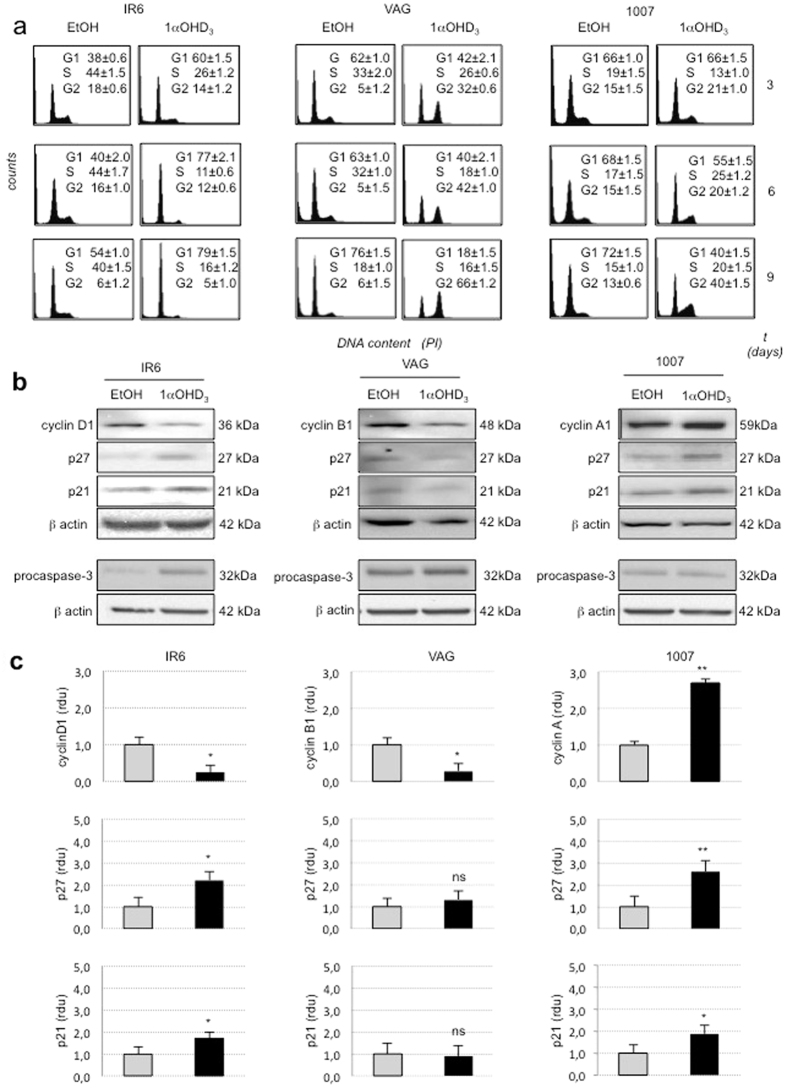
Cell cycle analysis of tumorigenic melanoma cell lines with and without vitamin-D_3_ exposure. (**a**) Fluorescence-activated cell sorting (FACS) of IR6, VAG and 1007 cell lines treated for 3, 6, and 9 days with (1α-OH-vitD_3_) or without (CTR) 1α-OH-vitD_3_ 20 ng/ml (5 × 10^−8^ M), Cell cycle perturbation and redistribution of IR6, VAG and 1007 melanoma cells in presence of vitamin-D_3_ is demonstrated. Row data regarding cell distribution (%) in G1, S, and G2 phases are shown in detail. (**b**) Western blot analysis to support FACS data, showing modulation of cell-cycle key regulatory molecules expected to be specifically involved in G1 and G2 blocks, performed on total cell lysates from IR6, VAG and 1007 melanoma cells after 72 h of vitamin-D_3_ treatment. In the bottom of panel B the lack of procaspase-3 cleavage is shown to demonstrate the absence of apoptosis in vitamin-D3 treated cells. (All the experiments were performed in triplicate; figure derived from a cropped gels/blots for semplification) (**c**) Densitometric analysis of cyclins (D1, A,B), p27 and p21 protein bands shown in panel B. Each band intensity was first normalized to the corresponding band of internal control (β-actin). Bands obtained from vitamin-D treated and untreated cells were compared each other, normalizing those derived from untreated cells to 1. The band intensity is represented in the graph as relative densitometric unit (rdu). Data are means ± SD of the relative band intensity from three independent WB experiments. Standard deviation is shown (*Statistical analysis using t-test: ns* = not significant; *p < 0.05; **p < *0.01*).

1α-OH-vitD_3_ induced cell cycle arrest in all melanoma cell lines, acting at different levels. IR6 melanoma cells were blocked in G1-phase (60% *vs* 38% of the control cells) after 72 hrs of treatment. The effect was persistent at day 9 (79% *vs* 54%) indicating that melanoma cells were unable to recover in the presence of 1α-OH-vitD_3_ (Fig. 5a).

VAG melanoma cells were blocked in G_2_ phase. Such a perturbation of the cell-cycle was observed after 72 hrs of treatment (32% *vs* 5%) but it was much more evident on day 9 (66% *vs* 6%) (Fig. 5a).

In 1007 melanoma cell line 1α-OH-vitD_3_ induced cell accumulation in the proliferative compartments (S plus G2 phase) at days 6 and 9, compared to the control cells. This was associated with a loss of cells in G1 phase (55% *vs* 68% at day 6 and 40% *vs* 72% at day 9) (Fig. 5a).

The sub-G1 peak (apoptosis peak) was not observed in these experiments. Lack of apoptosis was also demonstrated by the absence of PARP-1 and procaspase-3 cleveage in western blot analysis of cell lysates from 1α-OH-vitD_3_ treated melanoma cells (data not shown and Fig. 5b). Altogether these findings, suggest that 1α-OH-vitD_3_ Induces cell-cycle perturbation and arrest of melanoma cell proliferation in G1 or G2 phase, whereas apoptosis (or cell death for toxicity) was not observed in this experimental condition.

### 1α-OH-vitD_3_ Modulates the Expression of Cell-Cycle Regulatory Molecules

To further analyze the cell-cycle perturbation induced by 1α-OH-vitD_3_ in melanoma cells, the expression of candidate cell-cycle regulatory molecules was evaluated by western blot analysis (Fig. 5b,c). Cell-cycle arrest in G1 phase, induced by 1α-OH-vitD_3_ in IR6 melanoma cell line correlated with an increased expression of the cyclin-dependent kinase inhibitors p21 and p27, and down-regulation of cyclin-D1. On the other side, the arrest in G2 phase observed in VAG was associated to a slight decrease in cyclin-B1 expression level (Fig. 5b,c). In 1007 melanoma cell line 1α-OH-vitD_3_ induced an increased expression of cyclin-A1 with slight up-regulation of p21 and p27 according to the accumulation of melanoma cells in the proliferative compartment (S plus G2 phase) (Fig. 5b,c). These findings indicate that cell cycle perturbation induced by 1α-OH-vitD_3_ in melanoma cells involves the recruitment of regulatory molecules and pathways, which are specific for each melanoma cell line. However, the observed vitaminD-mediated effects on cell cycle, invariably converge toward an impairment of melanoma cell proliferation.

Interestingly the observed cell cycle inhibition seems to be independent by the BRAF status (see Supplementary Table S2 also).

### Long-term Systemic Administration of 1α-OH-vitD_3_ Inhibits Melanoma Cell Growth *in Vivo*

**Figure 6:**
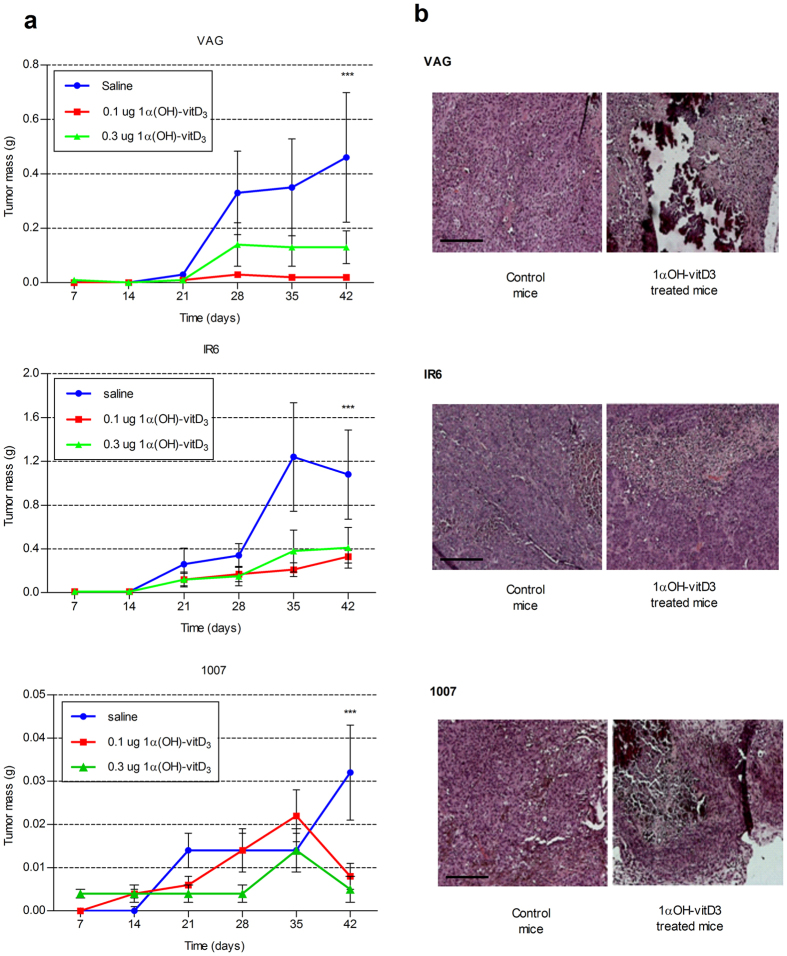
Effects of long-term systemic administration of 1α-hydroxycholecalciferol on melanoma growth *in vivo.* (**a**) Tumor mass variations at the end of vitamin-D_3_ treatment *in vivo* (day 42 from melanoma cells injection). Each point represents the average of values of four xenografted melanomas. Values at day 42 were directly determined at autopsy. (S.D. is reported on the top of each point. Statistical analysis at 42 days using ANOVA and Bonferroni post-hoc test: saline vs treated mice ***p < *0.001*). (**b**) Representative panel of histological slides showing tumor necrosis and dystrophic calcifications in the vitamin-D_3_ treated residual melanoma xenografts collected at autopsy. Melanoma xenografts from animals injected with saline solution alone, were used as comparative controls. (*Formalin-fixed and paraffin embedded histological preparations; haematoxylin-eosin staining; scale bar* = *200* *μm*).

*In vivo* experimental models of human melanoma xenografts were finally used for studying the long term effects induced by 1α-OH-vitD_3_ on melanoma growth. Daily administration of 1α-OH-vitD_3_ (see Material and Methods section for detail) markedly inhibited melanoma growth *in vivo.* The biological effect observed after 42 days from melanoma cells injection was statistically significant (Fig. 6a). The antiproliferative effect of 1α-OH-vitD_3_ on melanoma xenografts was finally confirmed at autopsy. The final histological evaluation of residual explanted tumours showed large areas of tumor necrosis with dystrophic calcifications in melanoma xenografts derived from vitamin D treated mice, but not in tumors explanted from control animals (Fig. 6b). A slight hypercalcemia with 5–10% body weight loss was observed in all of 1α-OH-vitD_3_ treated mice at the end of the experiment but not in the control animals (data not shown). These findings might suggest the occurrence of potentially harmful side effects of long-term administration of 1α-OH-vitD_3_. However, at the end of the experiment all the animals were in good health. No histological alterations in kidney, liver, lung or other organs were observed at the final histological examination (data not shown).

Interestingly, 1α-OH-vitD_3_-mediated modulation of expression of cell cycle regulatory molecules, previously observed *in vitro*, was also detected *in vivo* at immunohistochemical level (Supplementary Figure S3 and data not shown). These findings further support the notion that vitamin D active metabolites may trigger inhibitory signals in melanoma cells, which impair cell proliferation and tumor growth.

## Discussion

Vitamin D has pleiotropic effects of relevance to cancer with potential therapeutical implications in oncology^7^,^8^,^9^,^10^. By using experimental models of human melanoma we demonstrate that 1α-OH-vitD_3_ consistently impairs melanoma cell proliferation and tumor growth *in vitro* and *in vivo*. The possibility to take advantage of the vitamin D-mediated biological effects for treating melanoma patients is further supported by epidemiological and clinical evidence: i) Large observational studies show a significant association between low serum levels of 25-OH-vitD_3_ and increased cancer incidence^11^,^12^,^13^,^14^,^15^,^16^. Meta-analysis of case-control and cohort studies demonstrate that individuals with 25-OH-vitD_3_ ≥ 33 ng/mL (82 nmol/L) had a 50% lower incidence of colorectal cancer^14^; ii) Interestingly and in line with our work hypothesis, patients with early stage non small cell lung carcinomas with normal 25-OH-vitD_3_ serum levels and high vitamin D intake, had improved overall and recurrence-free survival^16^ iii) Preclinical data also indicate that active metabolites of vitamin D play as potential anticancer agents in different tumor models^10^,^17^,^18^,^19^,^20^,^25^,^26^,^27^. We demonstrate here that malignant melanoma represents a potentially responsive target to vitamin D. Although mutational analysis of VDR gene, CYP27A1, CYP27B1 and CYP24 A1 genes may be required before to consider a vitamin D treatment, relevant mutational events, which impair melanoma cells sensitivity to D-hormone, seem to be sporadic. The scenario of vitamin D –mediated biological effects is very complex to be analyzed in detail. Vitamin D active metabolites regulate important biological processes *via* genomic and non-genomic effects. Non genomic effects are in part mediated by the increase of free cytosolic calcium levels, whereas genomic effects require the binding to VDR, the predominant nuclear receptor protein expressed on target cells and tissues. VDR binds vitamin-D ligand with high affinity, resulting in heterodimerization with retinoid X receptor (RXR) and in zinc-finger-mediated binding to vitamin D responsive elements (VDREs), which modulate transcription of the target genes^26^,^27^,^28^,^29^,^30^. As a general feature all the patients-derived melanoma cell lines we tested were sensitive to 1α-OH-vitD_3,_ although the occurrence of vitamin D_3_ resistant melanoma cell lines has been reported^31^. Vitamin D_3_ mediated-arrest of cell proliferation and modulation of the expression repertoire of p21, p27, cyclin-D1 and likely of other cell-cycle key regulatory molecules, seem to be common biological events in melanoma experimental models used in this study. Interestingly this scenario has been observed also in different cell systems^26^,^27^,^28^,^29^,^30^. It is noteworthy that the promoter regions of specific cell cycle regulatory genes posses VDREs, which modulate gene transcription in response to vitamin-D/VDR binding^10^,^32^. This mechanism, which demonstrates a direct functional role of vitamin D in regulating the cell cycle, corroborates our findings.

By using *in vitro* experimental models we show that 1α-OH-vitD_3_ induces cell cycle arrest of IR6 melanoma cells in G1 phase, whereas VAG melanoma cells were blocked in G2 phase and 1007 melanoma cells accumulate in the proliferative compartment (S plus G2 phases). Such a cell-type specific perturbation of cell cycle was recoverable up to 6 days of vitD_3_ treatment, after that time melanoma cells proliferation was definitively impaired. Early VitD_3_ removal by the culture medium, infact, restored melanoma cell proliferation. This effect was quite evident in all melanoma cell lines considered in this study, with differences related to the cell-proliferation rates and/or cell-type specific biological features. A detailed analysis of modulation of cell cycle regulatory molecules in these experimental conditions represents an interesting field of research to be pursued in the near future.

Furthermore we finally demonstrate that a relatively long systemic treatment with 1α-OH-vitD_3_ also inhibits melanoma growth *in vivo*.

The differences in 1α-OH-vitD_3_ mediated-biological effects observed in melanoma cell lines may be explained, at least in part, by the presence of specific molecular alterations occurring in each specific tumor (i.e BRAF and KIT mutations, p53 status etc.). In each specific case, in fact, 1α-OH-vitD_3_ could activate different pathways of cell response with different functional consequences on cell-cycle^33^. Interestingly, the melanoma cell lines considered in this study included tumors bearing BRAF^V600E^ mutation (IR6 and FOR) and BRAF wild-type (VAG, 1007, MUL, LOJ), but the observed 1α-OH-vitD_3_ mediated-biological effects on cell proliferation and tumor growth occurred independently by the BRAF *status.* The potential functional role of vitamin-D in triggering tumor cell differentiation and/or senescence also deserves consideration. It is well known that normal melanocytes have a dendridic shape and that a E-chaderin plays a pivotal role in melanocyte-keratinocyte interaction in normal conditions. After neoplastic transformation this phenotype is generally lost. In the present study we observed that 1α-OH-vitD_3_ induced morphological changes (dendritic processes) and E-chaderin expression in melanoma cells, suggesting a pro-differentiative activity. This interesting effect has been already observed in a different cell system^7^. Further experiments will be carried out to deeply investigate this important issue.

Finally, although the value of normal serum level of 25-OH-vitD_3_ has been recently revisited, the effective vitamin D intake for tumor prevention and therapy are not defined yet^23^,^34^,^35^. The discovery of insufficient 25-OH-vitD_3_ serum levels (10–30 ng/mL) in almost all melanoma patients analyzed in this study, opens the question on the effects that restored serum levels of D-hormone may potentially have on the overall and recurrence-free survival and eventually on melanoma prevention. Although a direct correlation between 25-OH-vitD_3_ serum levels and clinical-pathological prognostic parameters could not be found in our cohort of patients, because of the low number of cases in each specific subgroup, it is intriguing that 5 out of 6 melanoma patients with sufficient vitamin-D serum levels at time of surgery had *in situ*/microinvasive melanomas. Moreover, a direct correlation between 25-OH-vitD_3_ serum levels and melanoma thickness has been reported in some clinical studies that fully support our work hypothesis^36^,^37^,^38^,^39^.

In conclusion this preclinical study provides the biological and clinical rationale for using vitamin D in melanoma adjuvant therapy.

What remains to be defined is if restored 25-OH-vitD serum levels (30–76 ng/mL) will be sufficient to improve the overall and disease-free survival of melanoma patients with high risk of tumor progression.

The intriguing possibility to use D-hormone in association with the most recently proposed molecular targeted therapies for treating metastatic melanoma, also deserves consideration. Anti-BRAF and anti-KIT inhibitors, in fact, finally produce functional impairment of down-stream molecules that regulate cell proliferation (cell cycle) and survival, some of which are direct targets of vitamin D^10^,^40^,^41^,^42^.

Targeting the aforementioned oncogenic molecules has been reported to induce important objective responses in patients with advanced melanoma. However the occurrence of a relatively rapid drug resistence, as well as the paradoxical activation of downstream molecules provides the rationale for exploring a combined therapeutic approach^43^,^44^,^45^. In this context the demonstrated vitamin D-mediated antiproliferative effects may be oncologically relevant.

Concluding, the antiproliferative activity of 1α-OH-vitD_3_ in experimental melanoma models *in vitro* and *in vivo*, together with the discovery of 25-OH-vitD_3_ insufficiency in almost all melanoma patients at time of surgery, provide the biological and clinical rationale for using vitamin D in melanoma adjuvant therapy, alone or in association with other therapeutic options. Long-term randomized clinical trials will be necessary for demonstrating, definitively, the real impact of vitamin D on melanoma patients’ survival.

## Methods

### Melanoma Patients, Valuation of 25-OH-vitamin-D_3_ Serum Levels

105 melanoma patients were checked for 25-OH-vitD_3_ (the active measurable vitamin D metabolite) serum levels at time of diagnosis. Patients were 43 male and 62 female, age ranging from 28 to 87 years (median age 53.7), 49 of them had high risk cutaneous melanoma (Breslow’s thickness >0.75 mm).

Data regarding the basal serum levels of 25-OH-vitD_3_ in 101 matched (for sex and age) healthy individuals (blood donors) from the same community, were anonymously obtained from the blood bank at Saint’ Andrea Hospital and used as comparative control. The clinical features of melanoma patients object of this study are summarized in Supplementary Table S1. 25-OH-vitD serum levels were determined by using the fully automated chemiluminescence method Liaison 25OHD assay system (Dia-Sorin, Saluggia VC, Italy) and classified according to the recent literature^23^. Briefly 25-OH-vitD_3_ serum levels <10 ng/mL were considered ‘deficient’; >10 to 30 ng/mL ‘insufficient’; and >30 to 76 ng/mL (75 to 190 nmol/L) were considered ‘normal’.

### Melanoma Cell lines, phenotipic and molecular characterization

Metastatic melanoma - derived cell lines (VAG, FOR, IR6, 1007, MAR, and LOJ) were established and phenotypically characterized at the Immunology Laboratory, National Cancer Institute Regina Elena of Rome, Italy. Most of these cell lines were previously described^46^,^47^,^48^,^49^,^50^,^51^. Cell lines were regularly checked immunocytochemically for the expression of specific melanoma markers (Mart-1, HMB-45, S-100) before each experimental procedure (data not shown). The expression of vitamin D receptor (VDR) was analyzed immunohistochemically as reported below and the occurence of VDR polymorphism was also investigated. Briefly, three major restriction fragment lenghts polymorphism (RFLP) were investigated, in the exon 2 and 3′-region, respectively the TaqI, BsmI and FokI polymorphisms^52^,^53^. The FokI polymorphism results in the production of a VDR protein that is three amino acids longer with an increase of transcription levels^54^. The BsmI is a RFLP in intron 8 at the 3′ end of the VDR gene, which may influence VDR messenger RNA stability^55^. TaqI polymorphism is a RFLP at codon 352 in exon 9 of the VDR gene, that leads to a silent codon change from ATT to ATC, which both result in an isoleucine at codon 352^56^.

Melanoma cell lines (VAG, FOR, IR6, 1007, MAR, and LOJ) were also tested for the presence of BRAF (exon 15 V600E) mutations. Genomic DNA was extracted using standard protocol (Roche) and subjected to amplification of the target gene using the following primers: BRAF-F: 5′-CCTAAACTCTTCATAATGCTT-3′ and BRAF-R: 5′-ATAGCCTCAATTCTTACCAT-3′. Nucleotide sequence analyses were performed using BigDye Terminator Cycle Sequencing Ready reaction Kit (Applied Biosystems) and an ABI PRISM 310 Genetic Analyzer (Applied Biosystems). The phenotypical and molecular features of melanoma cell lines used in this study are summarized in Supplementary Table S2.

### RT-PCR, Western Blot and Immunohistochemical Analysis

Total RNA extraction and cDNA synthesis were previously described^55^. PCR reactions were performed in PCR buffer (10 mM Tris-HCl pH 8.4; 500 mM KCl; 50 mM MgSO4) with 0.2 μmol primers and 2U Platinum Taq DNA Polymerase (Invitrogen) in a final volume of 50 μl for 35 cycles (denaturation at 95 °C for 30 seconds, annealing at 48 °C–52 °C, according to different pair of primers, for 30 seconds and extension at 72 °C for 1 minute). The following human oligonucleotides were used as primers: Vitamin D Receptor (VDR) *for* 5′-CCAGTTCGTGTGAATGATGG-3′, *rev* 5′-GTCGTCCATGGTGAAGGA-3′; CYP27A1 (25-OHase) *for*: 5′-GGCAAGTACCCAGTACGG-3′, *rev*: 5′-AGCAAATAGCTTCCAAGG-3; CYP27B1 (1α-OHase) *for*: 5′-TGTTTGCATTTGCTCAGA-3′, *rev*: 5′-CCGGGAGAGCTCATACAG-3′; CYP24A1 (24-OHase) *for*: 5′-GCAGCCTAGTGCAGATTT-3′, *rev*: 5′-ATTCACCCAGAACTGTTG-3′. Integrity and equal loading of cDNA in the PCR reactions were checked by quantification of 18S mRNA levels.

Mouse monoclonal antibodies (mAbs) to p21, p27, Cyclin-D1, E-cadherin, and human VDR NR1/1 (Space Import & Export, Milan, Italy); goat polyclonal antiserum to Cyclin-B1, rabbit polyclonal antiserum to Cyclin-A1 (Space Import & Export), mouse mAbs to β-actin (Santa Crutz Biotechnology, CA, USA) and rabbit polyclonal procaspase-3 antibody sc-7148 (Santa Cruz Biotechnology) were purchased and used in immunochemical assays according to the manufacturer’s instruction. Immunohistochemical analysis on formalin fixed and paraffin embedded histological samples, as well as western blot analysis, were previously described^57^,^58^,^59^.

### Melanoma Cell Cultures and Vitamin D Treatment *in Vitro*

For *in vitro* experiments 1α-hydroxycho-lecalciferol (1α-OH-vitD_3_) (Dediol^®^ Sanofi-Aventis SpA, Milan, Italy, or Diseon^®^ Teva Italia Srl, Milan, Italy) was added to the culture medium at final concentration of 20 ng/mL (5 × 10^−8^ M). Drug concentration was selected after preliminary dose-response experiments *in vitro* (data not shown). Briefly, 3 × 10^5^ cells were seeded in six-well plate (Falcon Labware, Oxnard, CA USA) in complete RPMI-1640 culture medium, supplemented with 2% FBS. After 24 hours 1α-OH-vitD_3_ or solvent alone (0.1% ethanol) were added to the culture medium. The medium was replaced every 48 hours (in both treated and control cell lines) to guarantee a constant presence of fresh vitamin D. Cells were finally collected at 3, 6, 9 and 18 days of treatment (depending by the experiment) and used as targets for proliferation assays and FACS analysis as reported below. Similar experiments were performed by using the synthetic active derivative of vitamin-D_2_ worded paricalcitol (Zemplar ^®^ Abbott Srl, Latina, Italy) at concentration ranging from 0.08 μg/mL to 0.8 μg/mL^17^. Tumorigenic melanoma cell lines (1007, IR6, VAG) were finally selected for creating melanoma xenografts *in vivo* as reported below.

### Determination of Melanoma Cells Growth Rate *In Vitro*

Melanoma cells were seeded at concentration of 3 × 10^5^ cells/well in a six-well plate and maintained in complete RPMI 2% FBS with or without 1α-OH-vitD_3_ as reported above. Cell proliferation was determined by cell counting in a Burker camera after 3, 6, 9 and 18 days of vitamin D treatment, depending by the experiment.

To achieve melanoma cell cultures for 18 days, melanoma cells growing at confluence were split at appropriate time (according to the doubling time of each cell line: T_d_IR6 = 36 hrs, T_d_VAG = 48 hrs and T_d_1007 = 72 hrs) by using a PBS solution containing trypsin 0.05% and EDTA 0.02% (Gibco). All the experiments were performed in triplicate. In a different set of experiments the MTS assay (colorimetric tetrazolium salt assay) was also used for evaluating cell growth rate *in vitro*. Cells were cultured at density of 5 × 10^4^ cells/well in flat-botton 96-well plates. Vitamin-D treatment *in vitro* was performed with 1α-OH-vitD_3_ at final concentration of 20 ng/mL (5 × 10^−8^ M) or paricalcitol at concentration ranging from 0.08 μg/mL to 0.8 μg/mL. After 3 days of treatment CellTiter 96^®^ Aqueous One Solution Reagent (Promega, Madison, WI, USA) was added to each well according to the manufacturer’s instructions. Cell viability was determined by measuring the absorbance at 490 nm using a 550 BioRad plate-reader (Bio-Rad, Hertfordshire, UK).

### Cell cycle profile analysis by flow cytometry (FACS analysis)

Melanoma cells adjusted to reach 60–70% confluence at the time of FACS analysis were collected after 3, 6, and 9 days of 1α-OH-vitD_3_ treatment, washed in phosphate buffered saline solution (PBS) pH 7.4 and fixed in 50% acetone/methanol (1:4 v/v) in PBS for 1 hour. Cells were re-suspended in a DNA staining solution containing propidium iodide (10 mg/ml^−1^) and RNAse (1.8 units μl^−1^) for 30 minutes and finally analyzed on a FACScan (Becton Dickinson, Immunocytomety System, Mountain View, CA, USA). Twenty thousand events *per* sample were registered. All the experiments were performed in triplicate.

### Melanoma Xenografts and 1α-OH-vitD_3_ Treatment *in Vivo*

36 pathogen-free Balb-c nude *(nu/nu)* mice, 4–5-weeks-old (Charles River Breeding Laboratories, USA) and tumorigenic human melanoma cells lines 1007, IR6 e VAG were used for establishing melanoma xenografts *in vivo*^47^,^48^. 36 mice were organized in 9 groups in cages of 4 animals each (12 mice for each melanoma cell line) and fed with standard diet and water *ad libitum*. One week after subcutaneous injection of melanoma cells (5 × 10^6^ cells/mouse) mice were treated every other day, for a total of six weeks, with 1α-hydroxycholecalciferol *via gavage,* according to the following protocol: *Groups 1 control mice:* saline solution only (200 μl/mouse); *Groups 2*: 1α-OH-vitD_3_ 4 I.U. (0.1 μg/200 μl volume/mouse); *Groups 3*: 1α-OH-vitD_3_ 12 I.U. (0.3 μg/200 μl volume/mouse). Mice in groups 2 received a total of 80 I.U. of 1α-OH-vitD_3_ in 42 days of treatment, whereas mice in groups 3 got 240 I.U. Tumor size was measured every 3 days and tumor weight (gm) was calculated as previously described^47^. Animal health conditions were monitored daily all along the experiment.

Calcium serum level was measured in each animal at the beginning and at the end of the experiment. Mice were sacrificed after 6 weeks of treatment. Explanted organs and residual tumors were processed for conventional histology and immunohistochemistry.

### Statistical Analysis

*In vitro* experiments have been repeated 3 times and the results obtained are presented as means ± standard deviation (SD). Significant changes were assessed by using Student’s t-test for unpaired data, two-way Anova and Bonferroni post-hoc tests for time and concentration effects (GraphPad Prism Software, version 5.0) a *p values* < 0.05 were considered significant.

### Ethics statement

Melanoma cell lines used in this study were established at the Immunology Laboratory, National Cancer Institute Regina Elena of Rome, according to the Institutional ethical guidelines provided by the Italian Ministry of Public Health (IMPH). I*n vivo* animal experiments were performed at the Animal Facility of the National Cancer Institute Regina Elena of Rome, under the direct control of an independent veterinary staff. Animal experiments were performed according to the guidelines provided by the IMPH. The study was approved by the Institutional Animal Care and Use Committee and by the multidisciplinary Board of Physicians of the Saint’ Andrea Melanoma Working Group (SAMWG).

Blood samples and tissues from humans were collected at Saint Andrea University Hospital and used anonymously in full agreement with the guidelines provided by the Institutional Review Board (Prot. CE no. 8391/2013) and Helsinki Declaration. Dermatologists from SAMWG, which have the responsibility for patients’ follow-up, collected a written informed consent from all the melanoma patients considered in this study.

## Additional Information

**How to cite this article**: Spath, L. *et al*. Antiproliferative Effects of 1α-OH-vitD_3_ in Malignant Melanoma: Potential Therapeutic implications. *Sci. Rep.* **7**, 40370; doi: 10.1038/srep40370 (2017).

**Publisher's note:** Springer Nature remains neutral with regard to jurisdictional claims in published maps and institutional affiliations.

## Supplementary Material

Supplementary Figures and Table
