# Supplementary material for: Antiproliferative Effects of 1α-OH-vitD3 in Malignant Melanoma: Potential Therapeutic implications
Source: Sci Rep. 2017 Jan 11;7:40370. doi: 10.1038/srep40370 (PMC5225467; doi:10.1038/srep40370)
Supplement: Supplementary Figures and Table [file srep40370-s1.pdf]

Antiproliferative Effects of 1α-OH-vitD$_3$ in Malignant Melanoma. Potential Therapeutic implications

Lucia Spath[1,+], Alessandra Ulivieri[1,4+], Luca Lavra[1,4], Laura Fidanza [2], Marta Carlesimo[2], Maria Giubettini[1], Alessandra Narcisi[2], Emidio Luciani[1], Barbara Bucci[1], Daniela Pisani[3], Salvatore Sciacchitano[4] and Armando Bartolazzi[1,5]

[1]Pathology Research Laboratory, [2]Dermatology Unit and [3]Internal Medicine Sant'Andrea University Hospital, via di Grottarossa 1035, 00189 Rome, Italy.

[4]Laboratory of Biomedical Research, Niccolò Cusano University Foundation, via Don Carlo Gnocchi 3, 00166 Rome, Italy

[5] Molecular and Cellular Tumor Pathology Laboratory, Cancer Center Karolinska, Karolinska Hospital, CCK R8:04, S-17176, Stockholm, Sweden.

Running Title: Vitamin D and Melanoma

Keywords: melanoma, vitamin D, adjuvant therapy, tumor xenografts, 1α-hydroxycholecalciferol.

*__Corresponding author__

Armando Bartolazzi M.D., Ph.D. : Dept. of Pathology St. Andrea University Hospital, Via di Grottarossa 1035, 00189 – Rome, Italy
Phone: +39-0633775321; Fax: +39-0633775032;
E-mail: Armando.Bartolazzi@ki.se

[+] L.S. and A.U. contributed equally to this paper

Supplementary figure S1
Schematic representation of vitamin-D synthesis and metabolism

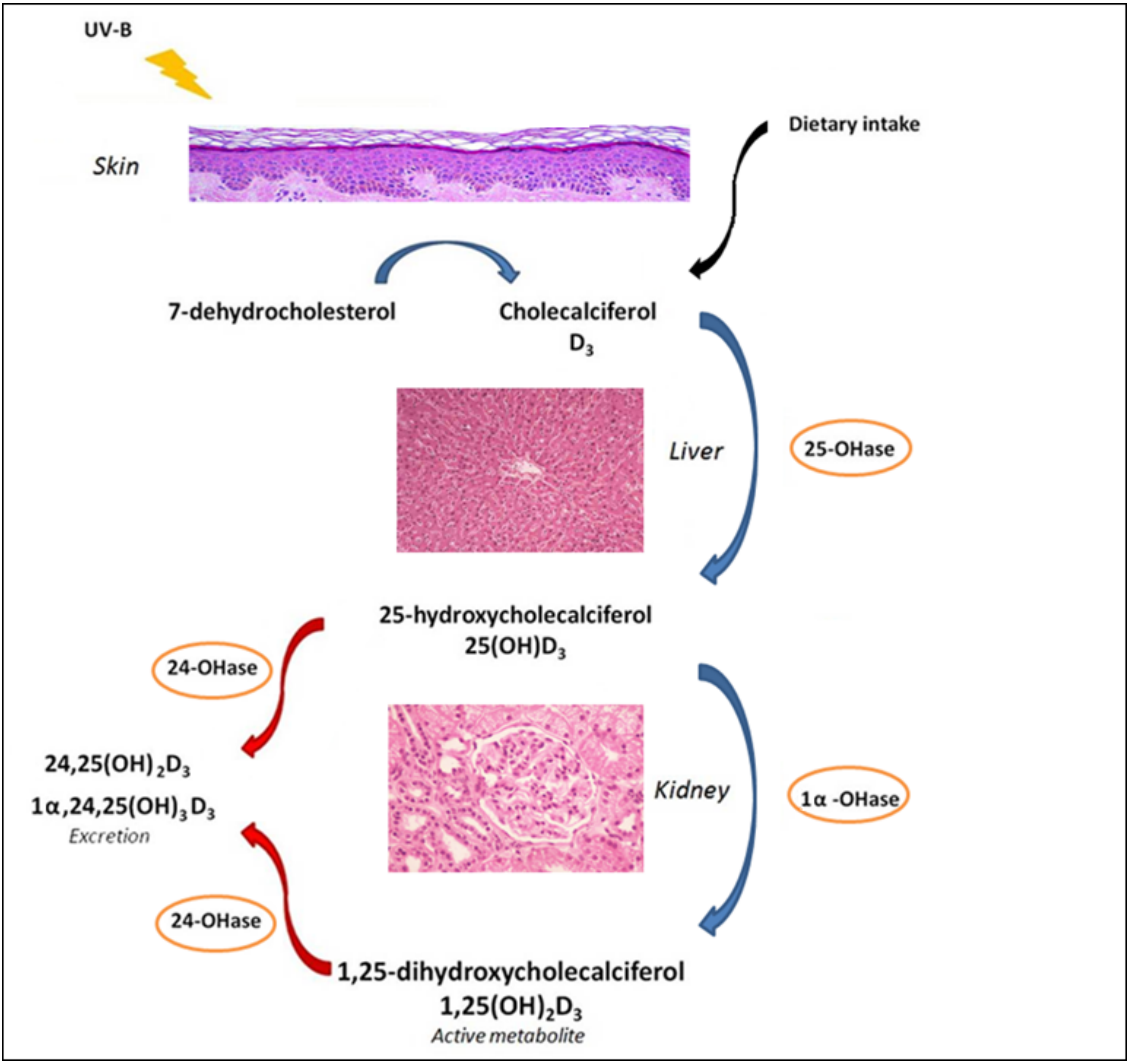

Supplementary figure S2
Human metastatic melanoma cell lines express VDR and the complete repertoire of transcripts for vitamin-D activation and catabolism.

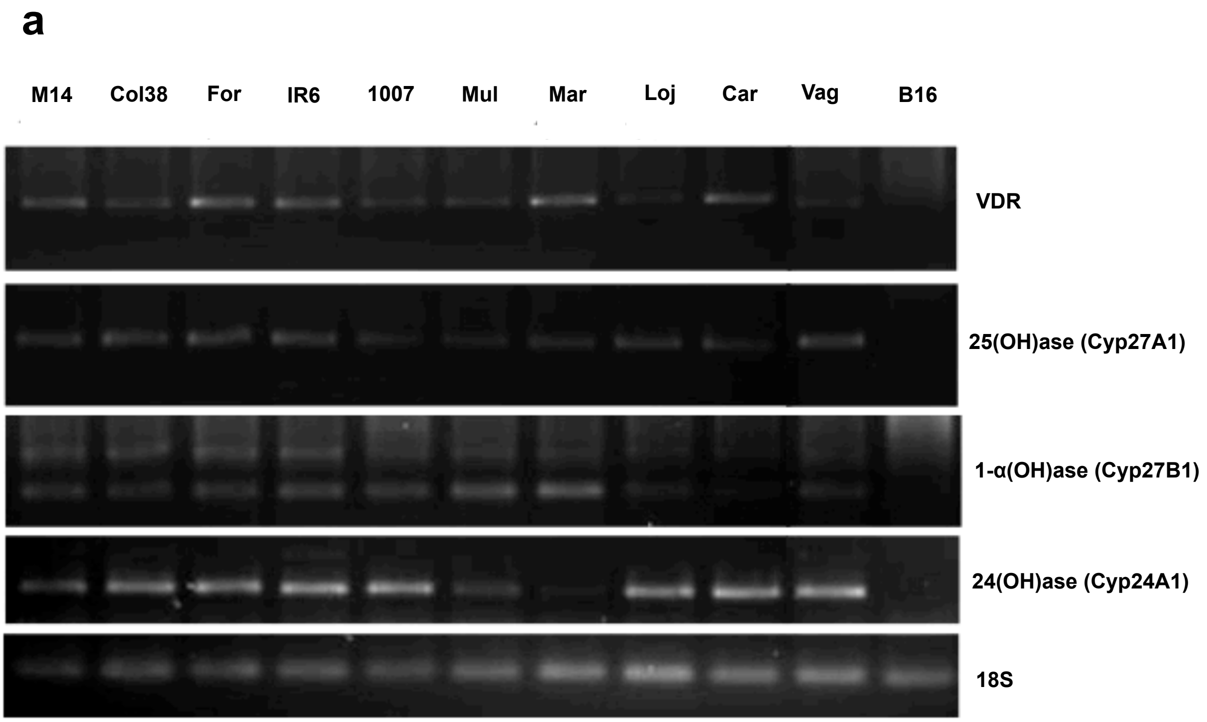

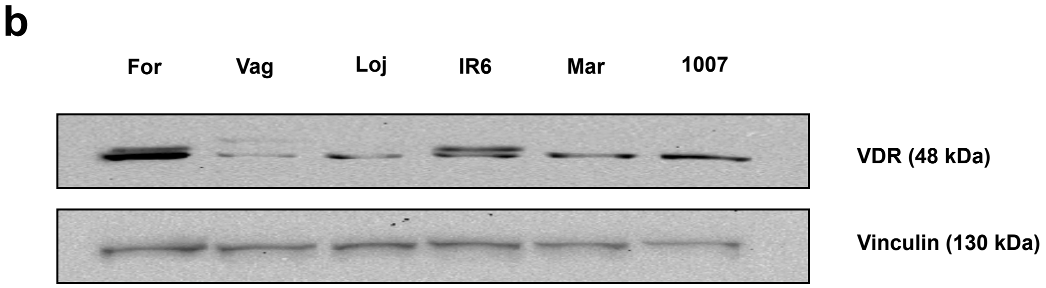

A) RT-PCR analysis performed on 10 human metastatic melanoma cell lines shows the expression of specific transcripts for the vitamin-D receptor (VDR), the activating enzymes 25-hydroxylase (25-OHase) and 1α-hydroxylase (1α-OHase), and the catabolic enzyme 24-hydroxylase (24-OHase). Murine B16 melanoma cell line was used as negative control and RNA 18S as loading control (see the material and methods section for detail). B) The expression of VDR in a panel of melanoma cell lines as evaluated in western blot analysis.

Suppementary figure S3
Vitamin-D3 induced modulation of expression of the cell cycle regulatory molecules in melanoma xenografts in vivo.

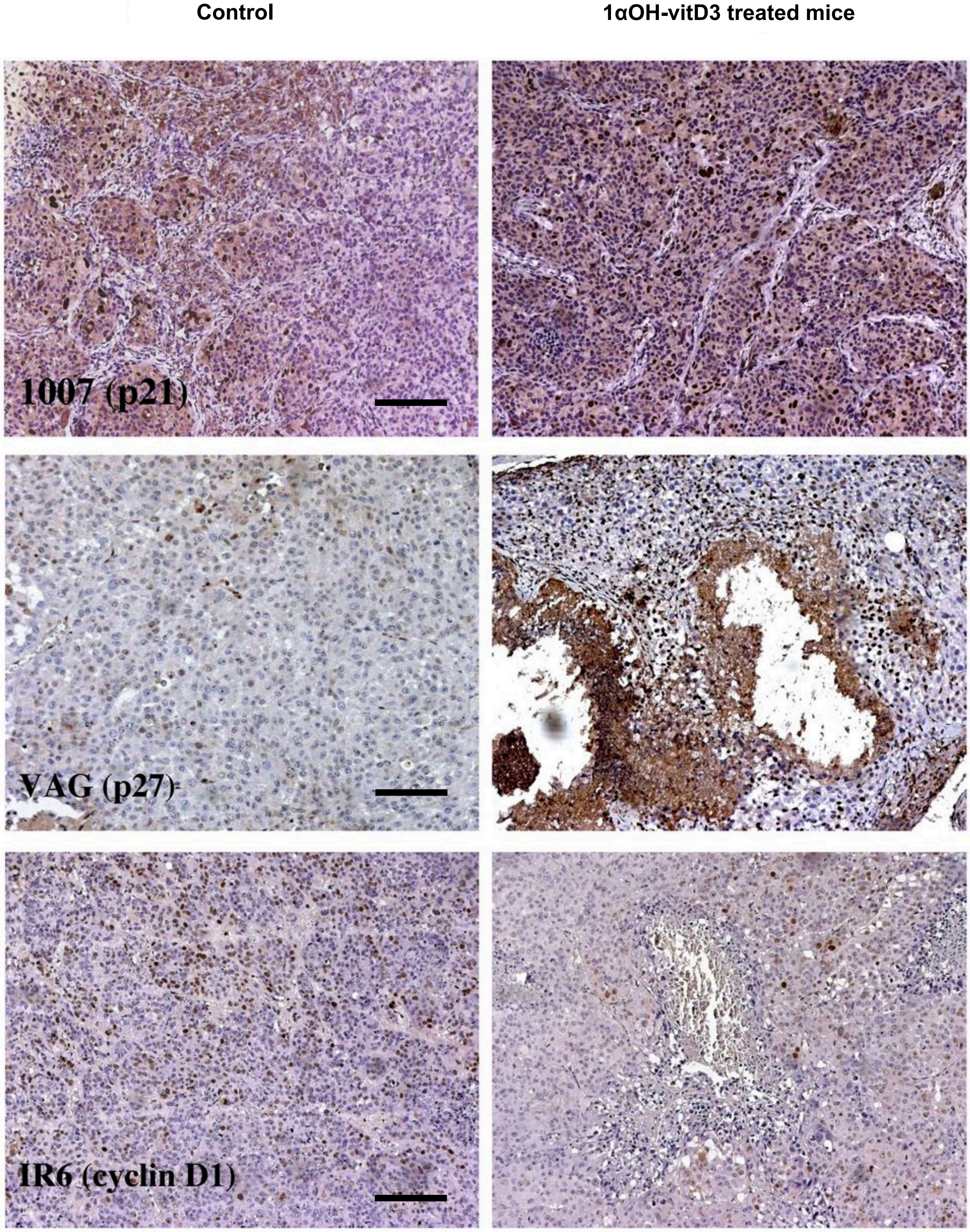

A representative panel of melanoma xenografts surgically excised after vitamin-D3 long-term systemic administration in vivo and processed for a comparative immunohistochemical evaluation of p21, p27, and cyclin-D1 expression. The sharp brown nuclei represent the positive cells. Up-regulation of p21 and a consistent down-regulation of cyclin-D1 were detected after vitamin-D3 treatment in 1007 and IR6 melanoma xenografts respectively. A slight increase of p27 was observed in VAG (Indirect Immunoperoxidase, LSAB-universal kit Dako on formalin-fixed and paraffin embedded tissues. Scale bar 200 μm.

Table S1

The clinical features of melanoma patients considered in this study

Patient code	Sex	Age	Melanoma Histology	Breslow thickness (mm)	Sentinel lymph node	25-OH-vitD3 serum levels (ng/mL)
1AF	F	44	SSM [a]	0,55	NA	28,4
2GSM	M	51	SSM	0,7	NA	30
3MC	M	33	SSM	0,65	NA	28,8
4LF	F	45	SSM	0,2	NA	15,8
5DG	F	70	SSM	0,2	NA	16,4
6RM	F	64	SSM	0,3	NA	27,6
7CS	F	32	SSM	0,2	NA	26,3
8CAT	M	49	SSM	0,2	NA	10,6
9GC	M	29	SSM	0,3	NA	29,8
10BM	F	62	SSM	0,3	NA	13.4
11GC	M	56	SSM	0,45	NA	15,3
12MG	M	67	LM [b]	0,55	NA	18,7
13CP	F	42	ALM [c]	0,8	pN0	15,3
14CR	M	36	SSM	0,8	pN0	15,5
15AR	M	57	SSM	0,85	pN0	16
16GA	M	58	SSM	0,9	pN0	17
17GA	M	42	SSM	1,1	pN0	8,7
18BG	F	39	SSM	1,1	pN0	17,7
19SM	M	82	SSM	1,1	NA	11,3
20MB	M	67	SSM	1,2	pN0	20,8
21AS	F	42	SSM	1,2	pN0	29,9
22RB	F	28	SSM	1,3	pN0	18
23LDM	M	70	NM [d]	1,3	pN0	17,8
24FG	M	53	NM	1,3	pN0	17,1

25GM	F	40	SSM	1,35	pN0	15
26IA	F	60	SSM	1,5	pN0	11,3
27IB	F	72	SSM	1,5	pN0	15,7
28NA	F	44	SSM	1,7	pN0	20,1
29AB	F	64	SSM	1,8	pN0	18,7
30CP	M	43	SSM	2,2	NA	8,4
31LQ	F	70	NM	2,2	pN0	13,6
32AMM	F	56	NM	2,4	pN0	16
33MM	M	71	NM	8	NA	26,8
34RD	F	46	Spitzoid M [e]	1.8	pN1	20
35FA	M	47	NM	2	pN1	27,7
36SM	F	55	SSM	2,7	pN1	10,8
37CB	M	61	NM	5	pN1	20
38MM	F	63	ALM	6	pN1	9
39RC	F	44	NM	6	pN1	29,8
40VM	M	32	NM	7	pN1	16,4
41SA	M	34	SSM	In situ	NA	18
42GA	F	71	LMM	In situ	NA	15,1
43FB	F	41	SSM	In situ	NA	17,6
**44MB**	**M**	**78**	**LMM**	**0,2**	**NA**	**42**
45CB	M	75	NM	5	pN0	20
46PB	F	87	LMM	In situ	NA	15,2
47AB	M	85	NM	9	pN0	19,4
**48MC**	**M**	**43**	**SSM**	**In situ**	**NA**	**35**
**49PC**	**F**	**48**	**SSM**	**In situ**	**NA**	**33,2**
50NC	F	30	SSM	In situ	NA	11,9
51DC	F	52	SSM	In situ	NA	25
52SC	F	55	SSM	0,15	NA	11,8
**53RC**	**F**	**46**	**NM**	**6**	**pN1**	**39,9**
54MC	M	44	SSM	0,3	NA	24,5
55FC	F	53	NM	3,5	pN1	15
56SC	F	49	SSM	In situ	NA	22,6

57GAD	M	86	ALM	In situ	NA	16.3
58LD	F	40	SSM	0,7	NA	23,2
59PD	M	56	SSM	0,5	NA	17
60MD	M	77	NM	2,5	pN0	14,5
61GD	F	42	SSM	1,5	pN0	23,1
62DLGD	M	62	ALM	In situ	NA	18
63EPD	F	42	SSM	0,2	NA	20
64GVD	F	70	ALM	0,3	NA	15,7
65DD	F	39	NM	3,5	pN0	12,4
66ECD	F	41	SSM	0,2	NA	15,1
67MCD	F	86	NM	3,6	pN0	12,6
68AF	F	44	SSM	0,55	NA	11,2
69SF	M	40	SSM	In situ	NA	20
70FF	F	40	SSM	0,3	NA	18,1
71NF	F	70	SSM	4,7	pN1	16,5
72OF	M	70	SSM	0,75	pN0	24,2
73GF	M	50	SSM	0,9	pN0	24,2
74GF	F	39	SSM	0,3	NA	7,92
75TG	M	47	SSM	0,7	pN0	9,03
76OG	F	40	SSM	0,5	NA	27,1
77AG	F	70	NM	4,6	pN1	21,8
78IG	F	67	SSM	In situ	NA	27,8
79ML	M	44	SSM	2,8	pN1	11,7
80LL	M	29	SSM	0,55	NA	16,2
81SL	F	66	SSM	1,58	pN0	22
82AL	F	54	SSM	0,2	NA	14
83AM	F	68	SSM	0,3	NA	10,8
84SM	F	66	SSM	2,7	pN0	10,8
85FPM	M	57	NM	3	pN0	11,3
86EM	M	46	SSM	In situ	NA	20,5
87MM	M	74	SSM	0,3	NA	14,7
88AJM	M	53	SSM	0,3	NA	9,34

89RM	F	78	SSM	1,4	pN0	22
90MN	F	70	LMM	In situ	NA	12,5
91GAN	M	46	NM	4	pN1	13,8
92EO	F	47	SSM	0,7	pN0	19,8
**93MP**	**F**	**47**	**SSM**	**In situ**	**NA**	**31,1**
94EMP	F	50	SSM	1,7	pN0	25,9
95RR	M	69	SSM	0,2	NA	28,3
96MR	F	37	SSM	0,8	pN0	20,1
97ER	F	71	SSM	1,5	pN0	15,8
98AR	M	76	SSM	1,3	pN1	31,9
99PS	F	30	SSM	0,1	NA	28,4
100EPS	F	47	SSM	0,45	NA	24
101CS	F	41	SSM	In situ	NA	21
102LT	F	85	SSM	In situ	NA	29,8
**103GPV**	**M**	**36**	**SSM**	**In situ**	**NA**	**30,2**
104SV	F	42	SSM	0,1	NA	7
105MS	F	60	SSM	0,2	NA	5,76

Legend

[a] SSM=Superficial Spreading Melanoma;

[b] LM= Lentigo Maligna Melanoma;

[c] ALM=Acral Lentiginous Melanoma;

[d] NM= Nodular Melanoma;

[e] Spitz M =Spitzoid Melanoma;

NA= Not available;

pN0 = negative sentinel lymph node at histology;

pN1=metastatic sentinel lymph node at histology.

Melanoma Patients with sufficient basal serum levels of 25-OH-vitD_3 are highlighted in bold

Table S2

VDR Polymorphism and Braf status in Melanoma Cell Lines

Cell line	VDR FokI cod.1 T-C	VDR BsmI intr. A-G	VDR TaqI exon 9 T-C	Braf
1007	HO	HE	HE	WT
IR6	HO	WT	HO	V600E
VAG	WT	WT	HO	WT
MUL	HO	WT	HO	WT
LOJ	HO	HE	HE	WT
FORM	HE	HE	HE	V600E

Legend: HO= homozygosis; HE= heterozygosis; WT= wild-type
